# Predictors of Severe Histological Chorioamnionitis and Associated Neonatal Outcomes in Term Intrapartum Clinical Chorioamnionitis: A Retrospective Cohort Study

**DOI:** 10.3390/medicina62050937

**Published:** 2026-05-11

**Authors:** Mariachiara Bosco, Simone Garzon, Chiara Simonetto, Beatrice Cattin, Elisa Ida Erbogasto, Benjamim Ficial, Carlotta Milocchi, Ricciarda Raffaelli, Laura Uccella, Massimo Franchi, Stefano Uccella

**Affiliations:** 1 Unit of Obstetrics and Gynecology, Department of Surgery, Dentistry, Pediatrics, and Gynecology, Azienda Ospedaliera Universitaria Integrata Verona, University of Verona, 37126 Verona, Italysimone.garzon@univr.it (S.G.);; 2 Neonatal Intensive Care Unit, AOUI Verona, 37126 Verona, Italy; 3 Emergency Department, EOC–Ospedale Regionale di Lugano, 6900 Lugano, Switzerland

**Keywords:** chorioamnionitis, fetal inflammatory response syndrome, meconium, neonatal sepsis, placental disease

## Abstract

*Background and Objectives*: Intrapartum clinical chorioamnionitis at term is a complication associated with adverse maternal and neonatal outcomes. We aimed to identify factors independently associated with severe histological chorioamnionitis in women with clinical chorioamnionitis at term and with severe histological chorioamnionitis accompanied by neonatal intensive care unit (NICU) admission, considered the most severe end of the spectrum. *Materials and Methods*: We retrospectively identified all women with a diagnosis of clinical chorioamnionitis during labor at term between 2017 and 2022. Maternal characteristics and maternal and neonatal outcomes were extracted from medical records. The study population was stratified by the presence or absence of histological chorioamnionitis. *Results*: Out of 12,332 women, 171 (1.4%) singleton pregnant women had an intrapartum diagnosis of clinical chorioamnionitis at term. A total of 96 (56.1%) were confirmed with severe histological chorioamnionitis. Thick meconium-stained amniotic fluid (MSAF) (OR = 5.88; 95% CI 1.12–30.86, *p* = 0.035) and advanced maternal age (OR = 1.14, 95% CI 1.01–1.29, *p* = 0.024) were independently associated with severe histologic chorioamnionitis. In women with severe acute histological chorioamnionitis, we observed a higher prevalence of neonatal NICU admission and longer NICU stay. Factors independently associated with severe histologic chorioamnionitis accompanied by adverse neonatal course (NICU admission) were maternal age, fetal tachycardia and thick meconium-stained amniotic fluid. *Conclusions*: The diagnosis of clinical chorioamnionitis has a low positive predictive value for intra-amniotic infection or inflammation. Factors such as maternal age, thick meconium-stained amniotic fluid and fetal tachycardia may help identify patients with underlying intra-amniotic infection and those at risk of worse perinatal outcomes. Improved identification may help avoid both over- and undertreatment and guide interventions aimed at preventing adverse neonatal outcomes.

## 1. Introduction

Clinical chorioamnionitis complicates around 4% of all pregnancies [1,2], with differences according to gestational age [3,4,5]. This condition is associated with adverse maternal outcomes (e.g., prolonged labor, cesarean delivery, postpartum endometritis, postpartum hemorrhage, intensive care unit admission, sepsis, death) [3,6,7] and neonatal morbidity and mortality, including long term sequelae such as cerebral palsy and other neurodevelopmental disabilities [8,9].

Chorioamnionitis is considered an ascending infection of the amniotic cavity [10,11] presenting with signs and symptoms of maternal systemic inflammatory response. Indeed, the diagnosis of clinical chorioamnionitis has been historically based on the presence of maternal fever associated with maternal and fetal tachycardia, uterine tenderness, foul-smelling discharge, or maternal leukocytosis [12,13].

However, several lines of evidence showed that only 54% of women with a diagnosis of clinical chorioamnionitis at term have an infection of the amniotic cavity, 15% do not have either infection or inflammation, and 24% have sterile intra-amniotic inflammation [10,14,15]. Therefore, the proposed criteria for clinical chorioamnionitis have low accuracy in identifying women with intra-amniotic infection, either confirmed by microbiological analysis or by histologic evidence [16,17].

An accurate identification of women in labor at term with clinical chorioamnionitis associated with intra-amniotic infection is key for correct patient management and to avoid over- and undertreatment. The aim of this study was to identify factors independently associated with severe histological chorioamnionitis in women with clinical chorioamnionitis at term. The secondary objective was to determine which factors were independently associated with severe histological chorioamnionitis accompanied by neonatal intensive care unit (NICU) admission, representing the most clinically significant end of the disease spectrum.

## 2. Materials and Methods

### 2.1. Study Population

This retrospective study was conducted over a five-year period (November 2017 to November 2022). Data were retrieved from a prospectively collected database including all 12,332 consecutive women who delivered in the labor and delivery unit of our University Hospital during the study period. This database was reviewed to identify patients with a diagnosis of intrapartum clinical chorioamnionitis. We included pregnant women at term (≥37 weeks) with singleton pregnancy. Women who delivered preterm or with multiple pregnancies were excluded. Data regarding demographic and obstetrics characteristics and outcomes were reviewed by a medical staff and neonatal data were extracted from NICU records by neonatologists.

The design, analysis, interpretation of data, drafting, and revisions conform to the Helsinki Declaration, the Committee on Publication Ethics Guidelines, and the Strengthening the Reporting of Observational Studies in Epidemiology (STROBE) Statement. Each patient signed the informed consent to allow the anonymized data collection and analysis for research purposes. Formal approval by the institutional review board was obtained.

### 2.2. Clinical Chorioamnionitis

Intrapartum clinical chorioamnionitis was defined as maternal fever plus at least one of the following findings: baseline fetal heart rate > 160 beats/min for ≥10 min, maternal white blood cell (WBC) count > 15,000/mm^3^ in the absence of corticosteroids, or purulent-appearing fluid coming from the cervix [18]. Maternal fever was defined as a tympanic temperature ≥ 37.5 °C confirmed on two measurements 30 min apart. This cut-off was chosen because no differences were found in the rates of confirmed chorioamnionitis among pregnant women with fever below or above 38 °C [19] and also because chorioamnionitis is not always associated with intrapartum fever [16,18,19]. Women with a diagnosis of intrapartum clinical chorioamnionitis were given antibiotic therapy as per our institutional protocol based on available guidelines: ampicillin 2 g iv every 6 h and gentamicin 2 mg/kg iv load followed by 1.5 mg/kg every 8 h.

### 2.3. Histologic Chorioamnionitis

The placenta of women with intrapartum clinical chorioamnionitis were systematically sent for pathological examination, meaning that histological evaluation was available for all included cases. Acute histologic chorioamnionitis was defined according to the Redline’s criteria. The term stage (I–III) refers to the progression of the process based on the anatomical region infiltrated by neutrophils while the term grade (1–2) refers to the intensity of the inflammatory process at a particular site. In the maternal inflammatory response, stage I lesions are defined by the presence of neutrophils within the chorion or subchorionic space. Stage II corresponds to neutrophilic infiltration of the chorionic connective tissue and/or amnion or involvement of the chorionic plate. Stage III represents necrotizing chorioamnionitis characterized by epithelial necrosis of the amnion. Grading reflects the intensity and distribution of neutrophilic infiltration in the placental membranes and chorionic plate. Grade 1 (mild–moderate) consists of scattered individual or small clusters of maternal neutrophils diffusely infiltrating the chorion laeve, chorionic plate, subchorionic fibrin, or amnion. Grade 2 (severe) is defined by the presence of at least three chorionic microabscesses, i.e., confluent aggregates of approximately 10–20 neutrophils, typically located between the chorion and decidua or beneath the chorionic plate. Grade 2 may also be assigned when a continuous band of confluent neutrophils more than 10 cells in thickness involves over half of the subchorionic fibrin or extends around one complete membrane roll [20]. In our study, histologic confirmation of chorioamnionitis was considered if acute placental inflammatory lesions stage II–III or grade 2 were observed [10,21,22,23]. The maternal host response to intra-amniotic infection can be evidenced by the presence of acute histologic chorioamnionitis at placental pathology [20], and severe histologic chorioamnionitis (Redline stage II/III or grade 2) has been shown to be more frequently associated with intra-amniotic infection. Evidence of fetal inflammatory response (i.e., funisitis) was also recorded as a sign of fetal response to intra-amniotic infection [10,22,24].

In our study, microbiological analysis (placental swabs for traditional culture) was performed by using sterile swabs to collect material from the amnion, the chorion, and the carefully exposed chorion–amnion interface. Microbiologic results were available only in 80% of cases, as placental swabs were performed according to institutional clinical practice at the discretion of the treating clinician. We also acknowledge that microbiological analysis by placental swab culture has important limitations due to the low sensitivity and the possible sample contamination after delivery; therefore, we did not use these results to assess the presence or absence of intra-amniotic infection.

### 2.4. Clinical Definitions and Management

Labor was diagnosed in the presence of regular uterine contractions (four in 20 min for a minimum of 1 h) and cervical modifications [25]. Prolonged premature rupture of membranes was defined as membrane rupture before the onset of labor and that had lasted for ≥24 h. Pregestational and gestational diabetes was defined according to the American Diabetes Association (ADA) guidelines [26]. Amniotic fluid was defined as clear, light, moderate, or heavy meconium-staining according to the amount of meconium [27]. Neonatal acidosis at birth was defined by an umbilical artery blood pH < 7.00 and base excess (BE) ≤ −12 mmol/L [28]. According to our institutional protocol, neonates at risk for infection are closely monitored in the first 48 h of life. Neonates developing signs or symptoms of infection are admitted to the neonatal intensive care unit (NICU), undergo blood sampling for complete blood count, C-reactive protein (CRP) assay, blood cultures, and receive broad-spectrum empirical antibiotic therapy (ampicillin 50 mg/kg iv every 8 h and gentamicin 4 mg/kg iv every 24 h). If the blood culture remains negative after 48 h, antibiotic therapy is suspended. Positive neonatal C-reactive protein (CRP) was defined as neonatal plasma concentration > 5 mg/L.

Among maternal outcomes, we defined maternal postpartum hemorrhage as an estimated blood loss of ≥500 mL and ≥1000 mL after vaginal and cesarean delivery, respectively. Blood loss was estimated using graduate bags plus gravimetric methods. Persistent postpartum fever was defined as postpartum maternal tympanic temperature ≥ 37.5 °C in two measurements at least 30 min apart after delivery.

### 2.5. Statistical Analysis

Continuous variables were assessed for distribution using visual inspection of histograms and the Shapiro–Wilk test. Normally distributed variables are presented as mean ± standard deviation (SD), and non-normally distributed variables as median and interquartile range (IQR). Categorical variables are presented as counts and percentages. Descriptive statistic was performed using the Student’s *t*-test or the Mann–Whitney U test for continuous variables and the chi-square or Fischer’s Exact test for categorical variables, as appropriate. The primary outcome was severe histological chorioamnionitis (HCA) confirmed on placental histopathological examination. The secondary outcome was severe histological chorioamnionitis accompanied by adverse neonatal outcome, defined a priori as neonatal intensive care unit (NICU) admission within the first 72 h of life.

Candidate predictors were prespecified based on clinical relevance and biological plausibility and the following were included: maternal age, gestational age at delivery (potential confounders), duration of the pre-labor rupture of membranes, amniotic fluid characteristics, intrapartum maternal temperature, maternal white blood cell count during labor, C-reactive protein level, fetal tachycardia (possible intrapartum predictors of intra-amniotic infection) and labor analgesia (management factor which can confound the diagnosis of clinical chorioamnionitis).

To identify independent factors associated with the primary outcome, multivariable logistic regression modeling was performed. Because the number of outcome events was limited relative to the number of candidate predictors and separation was possible, standard maximum-likelihood logistic regression was considered at risk of small-sample bias and unstable estimates. Therefore, bias-reduced penalized logistic regression was applied using median bias-reducing adjusted score equations, a Firth-type correction that mitigates small-sample bias while preserving an interpretable odds-ratio framework. Secondly, to evaluate factors associated with the secondary outcome (severe histological chorioamnionitis accompanied by NICU admission), a separate multivariable model was constructed using the same modeling strategy. Given the smaller number of events for the secondary endpoint, the number of candidate predictors was intentionally reduced a priori to avoid model overfitting. Predictor selection was guided by clinical relevance and by variables identified as most strongly associated with the primary outcome, as well as those considered clinically important for neonatal risk stratification. The same bias-reduced penalized logistic regression approach was applied. Missing data were handled using a complete-case approach for the variables included in the final multivariable model, so only observations with non-missing outcome and all included predictors contributed to the regression. This complete-case strategy was selected to ensure consistent adjustment across covariates within a single fitted model, acknowledging the reduction in effective sample size due to missingness in select predictors.

Model results are reported as adjusted odds ratios (ORs) with 95% confidence intervals (CIs) alongside two-sided *p*-values. Statistical significance was assessed at an alpha level of 0.05. Multicollinearity was assessed by inspecting correlations among continuous covariates and by calculating variance inflation factors (VIFs) for predictors included in the multivariable models. Analyses were performed using R (version 4.5.0; R Foundation for Statistical Computing, Vienna, Austria).

## 3. Results

A total of 12,332 women with singleton pregnancy at term delivered at our University Hospital between 2017 and 2022. We identified 171 women with a diagnosis of clinical chorioamnionitis. Of these, 96 (56.1%) were subsequently confirmed with a diagnosis of acute severe histological chorioamnionitis (stage II–III or grade 2). Acute funisitis was observed at pathological evaluation in 49 out of the 96 cases with acute severe histological chorioamnionitis while it was absent in all 75 women without it.

Maternal characteristics and maternal outcomes of women with clinical chorioamnionitis, stratified by the presence or absence of severe histological chorioamnionitis, are displayed in Table 1.

Among women with clinical chorioamnionitis at term, factors observed to be independently associated with acute severe histological chorioamnionitis in the multivariable logistic regression model were thick meconium-stained amniotic fluid (MSAF) (OR = 5.88; 95% CI 1.12–30.86, *p* = 0.035) and maternal age (OR = 1.14, 95% CI 1.01–1.29, *p* = 0.024) (Table 2).

Placental swab results were available for 83.0% (142/171) of cases. Among women with acute severe histological chorioamnionitis and with placental swab results available, placental swabs were positive in 16 patients (20%, 16/80), while we observed five positive results (8.1%, 5/62) among women without it (*p* = 0.047). Bacteria isolated by traditional culture in positive swabs (n = 16) were *Enterococcus* spp. (n = 7), *S. agalactiae* (n = 4), *Gram negative bacteria* (n = 3), *Morganella morganii* (n = 1) and *Brevibacterium* spp. plus *E. coli* (n = 1). Neonatal outcomes of women with clinical chorioamnionitis are displayed in Supplementary Table S1. Neonates born from women with severe histological chorioamniositis were more frequently admitted to NICU [38.9% (37/96) vs. 21.3% (16/95), *p =* 0.01] and they had a longer NICU stay [6 (2–8) vs. 2 (1–4) days, *p* = 0.008].

Maternal demographic and obstetric characteristics of the study population, stratified according to the presence of severe histological chorioamnionitis accompanied by an adverse neonatal course (NICU admission), are presented in Supplementary Table S2. Factors observed to be independently associated with acute severe histological chorioamnionitis accompanied by NICU admission in the multivariable logistic regression model were maternal age [OR 1.14, 95% CI 1.02–1.28, *p* = 0.015], fetal tachycardia [OR 3.10, 95% CI 1.02–10.59, *p* = 0.044] and thick meconium-stained amniotic fluid [OR 7.16, 95% CI 2.03–29.99, *p* = 0.0018] (Table 3). Variance inflation factor (VIF) analysis showed that the VIF values for all our included predictors in the multivariable regression analysis ranged from 1.1 to 1.2, which reflected minimal multicollinearity.

## 4. Discussion

### 4.1. Principal Findings

The main findings of this study are the following: (1) among women with a diagnosis of intrapartum clinical chorioamnionitis at term, thick meconium-stained amniotic fluid (MSAF) and maternal age were factors independently associated with an underlining severe histological chorioamnionitis; (2) neonates born from women with severe histological chorioamniositis were more frequently admitted to NICU and they had a longer NICU stay; (3) maternal age, fetal tachycardia and thick meconium-stained amniotic fluid were factors independently associated with severe histological chorioamnionitis accompanied by NICU admission (intended as the more severe end of the spectrum).

In our study we identified maternal age and thick MSAF as factors independently associated with severe histological chorioamnionitis (used as a proxy of intra-amniotic infection) [27,29,30,31,32]. The significance of MSAF and the degree of staining has been debated. It now seems clear that advanced gestational age at delivery and intra-amniotic infection/inflammation are factors associated with MSAF. Indeed, as gestational age progresses, the prevalence of MSAF increases, reaching 26.9% at 42 gestational weeks [33]. Similarly, a growing body of evidence supports the relationship between intra-amniotic infection and meconium-stained amniotic fluid. In patients with clinical chorioamnionitis at term, the proportion of microbial invasion of the amniotic cavity is higher among women with MSAF than those without meconium [34]. Bacteria in the amniotic fluid swallowed by the fetus are believed to stimulate fetal bowel peristalsis [28], and meconium in the amniotic fluid seems to enhance bacterial proliferation, acting as a growth factor against the bacteriostatic properties of the amniotic fluid [35,36]. Our study also showed that the proportion of neonates admitted to NICU and the length of NICU stay was significantly higher among women with severe histological chorioamnionitis than those without these findings at placental pathology. However, no other differences were found in neonatal outcomes. Notably, the prevalence of neonatal acidosis was similar in women with or without severe acute chorioamnionitis confirmed at placental pathology. This is consistent with the fact that clinical chorioamnionitis represents an inflammatory rather than a hypoxic stressor on the fetus, as also substantiated by the different patterns of cardiotocographic anomalies observed in fetuses exposed to inflammation when compared with those exposed to hypoxic events [37]. A similar proportion of neonates in the two groups developed signs suspicious of infection in the early neonatal period; the proportion of confirmed neonatal infection, neonatal sepsis, and neonatal antibiotic administration did not differ between women with or without severe histological chorioamnionitis. This might be partly due to the low prevalence of these outcomes, which may not have allowed us to detect a difference between the two groups. Moreover, the intrapartum antibiotics administration based on our institutional protocol may have had a confounding effect on both maternal and neonatal outcomes. However, the milieu of pro-inflammatory cytokines present in the amniotic fluid of women with intra-amniotic infection/inflammation can lead to fetal inflammation [38], which might result in poor neonatal outcomes even in the absence of documented neonatal infection, potentially explaining the outcomes observed in our study: higher NICU admission with a longer NICU stay. While the diagnosis of neonatal hypoxia and neonatal infection are straightforward from umbilical artery pH and neonatal microbiological exams, neonatal inflammation can only be identified if pro-inflammatory markers, such as IL-6 and other cytokines, are measured in umbilical cord blood, a test not routinely performed in clinical settings. However, there is a consistent body of evidence showing that fetal inflammation is associated with poor neonatal outcomes [39,40,41], and that early signs of fetal response to inflammation and also, in more severe cases, autonomic nervous system dysfunction might be observed at cardiotocography (e.g., fetal tachycardia and ZigZag pattern) [37,42].

Subsequently, to investigate the more severe end of the spectrum of intra-amniotic infection, we focused on possible factors independently associated with severe histological chorioamnionitis accompanied by neonatal NICU admission. Among women with clinical chorioamnionitis at term we observed that maternal age, fetal tachycardia and thick meconium-stained amniotic fluid were factors independently associated with this outcome. These results confirm that MSAF, particularly when thick, is a relevant risk factor for severe chorioamnionitis with a poorer neonatal clinical manifestation [35,36]. The observed association indicating that maternal age is an independent risk factor for our secondary outcome suggests that, among women with clinical chorioamnionitis, increasing maternal age is associated with a higher likelihood of underlying intra-amniotic infection complicated by NICU admission [2,43]. Lastly, our study showed that fetal tachycardia represents an additional independent risk factor of the more severe end of the spectrum (i.e., severe histological chorioamnionitis accompanied by NICU admission). In line with previous published literature, we know that fetal tachycardia is one of the earlier fetal responses to the intrauterine inflammatory milieu in cases of intra-amniotic infection/inflammation, which might also persist after birth, indicating that neonatal inflammatory involvement is present [37,44,45].

The results of our study confirmed that the diagnosis of clinical chorioamnionitis alone has limited specificity for identifying severe histological chorioamnionitis as only about half of the cases showed the histological findings consistent with intra-amniotic infection. However, we found that meconium-stained amniotic fluid (MSAF) and maternal age are significant risk factors for both severe histological chorioamnionitis and for the most severe end of the spectrum (i.e., those cases where placental findings are accompanied by NICU admission). Among women with clinical chorioamnionitis, fetal tachycardia represents an additional risk factor for intra-amniotic infection/inflammation requiring NICU admission after birth. These findings suggest that such cases might benefit from prompt antibiotic therapy, potentially even before a definitive diagnosis of clinical chorioamnionitis is made and that their neonates might also benefit from careful neonatal evaluation. Further research is warranted to identify early predictors of intra-amniotic infection/inflammation among women in labor at term. In particular, studies of cardiotocography (CTG) pattern analysis may provide additional insight, together with factors like MSAF, to improve the early detection of at-risk women.

### 4.2. Strengths and Limitations

The present study has several strengths. Despite its retrospective design, the data were prospectively collected in a structured database and systematically reviewed by trained medical personnel. Neonatal outcomes were extracted directly from dedicated neonatal records, spanning the NICU stay to discharge, thereby ensuring precise identification of adverse outcomes occurring in the early neonatal period.

This study also has limitations. The study population size might have limited the ability to identify differences between groups, particularly for outcomes with low prevalence. Only 80% of all included cases had microbiologic data (i.e., placental swab results), and no patients had the amniotic fluid cultured for microorganisms. Additionally, we did not manage to retrieve the exact time for antibiotic administration to assess if total therapy duration could have been a confounder in this study. Antibiotics may also attenuate the clinical expression of neonatal infection and modify early neonatal laboratory or clinical findings, thereby reducing the apparent association between histologic chorioamnionitis and overt infectious outcomes. Consequently, some neonates with true intra-amniotic infection exposure may have been clinically classified as unaffected. Therefore, the observed associations between histologic chorioamnionitis and neonatal outcomes should be interpreted with caution. We also acknowledge that half of our study was conducted during the COVID-19 pandemic, which might have impacted maternal and neonatal care [46,47]. Although maternal SARS-CoV-2 infection was not a predefined study variable and was not consistently documented for all cases, all women in our cohort were afebrile prior to labor onset and developed fever only intrapartum, making it less likely that maternal fever was attributable to SARS-CoV-2 infection.

Finally, this is a monocentric study with potential limitations in the generalizability of the results to settings with different obstetric or neonatal protocols.; however, obstetrics and neonatal protocols were uniform during the study period to allow homogeneity.

## 5. Conclusions

An accurate identification of women with intrapartum intra-amniotic infection/inflammation is key to avoiding overtreatment or undertreatment. The association of this condition with poor neonatal outcomes suggests that this subgroup of women and neonates warrants closer monitoring and possible earlier delivery to curb the impact of intra-amniotic infection/inflammation on neonatal outcomes.

## Figures and Tables

**Table 1:** Maternal demographic and obstetrics characteristics of the study population, overall and stratified according to the presence or absence of severe histological chorioamnionitis.

	Total N = 171	Without Severe Acute Histological Chorioamnionitis N = 75	With Severe Acute Histological Chorioamnionitis N = 96	*p* Value
**Maternal demographic and obstetrics characteristics**				
** Maternal age** (y; median, IQR)	31 (29–35)	31 (28–34)	32 (29–37)	0.10
** Pre-pregnancy BMI** (kg/m^2^; median, IQR)	22 (20–25)	22 (20–24)	22 (20–26)	0.27
** Nulliparity** (n, %)	150 (87.7)	65 (86.7)	85 (88.5)	0.71
** Previous cesarean section among multiparous** (n, %)	5 (18.5)	2 (14.3)	3 (23.1)	0.65
** Infertility treatments** (n, %)	14 (8.2)	4 (5.3)	10 (10.4)	0.23
** Gestational age at delivery** (weeks; median, IQR)	40.3 (39.6–41.1)	40.0 (39.1–40.7)	40.5 (39.7–41.2)	**0.008**
** Recto-vaginal GBS colonization** (n, %)	30 (17.8)	13 (17.3)	17 (18.1)	0.90
** Preexisting diabetes mellitus** (n, %)	1 (0.6)	0 (0.0)	1 (1.0)	1.0
** Gestational diabetes mellitus** (n, %)	22 (12.9)	11 (14.7)	11 (11.5)	0.53
**Labor characteristics**				
** Induction of labor** (n, %)	84 (49.1)	42 (56.0)	42 (43.8)	0.11
** Labor analgesia** (n, %)	127 (74.7)	53 (70.7)	74 (77.9)	0.28
** Duration of labor analgesia** (min; median, IQR)	480 (240–632)	455 (300–595)	490 (375–670)	0.13
** PROM** (n, %)	113 (67.7)	50 (68.5)	63 (67.0)	0.84
** Duration of PROM** (min; median, IQR)	593 (353–900)	570 (345–750)	660 (351–960)	0.45
** PROM ≥ 24 h** (n, %)	10 (9.1)	4 (8.0)	6 (10.0)	0.75
** Duration of labor, stage I** (min; median, IQR)	330 (180–480)	300 (170–397)	375 (202–506)	0.06
** Duration of labor, stage II** (min; median, IQR)	85 (45–126)	93 (45–146)	75 (42–120)	0.67
** Total duration of labor** (min; median, IQR)	410 (265–575)	390 (260–532)	449 (267–639)	0.17
** Amniotic fluid** (n, %)				**0.006**
** Clear**	88 (55.4)	47 (63.5)	41 (43.6)	**0.01**
** Thin meconium-stained**	15 (8.9)	8 (10.8)	7 (7.4)	0.44
** Moderate meconium-stained**	26 (15.5)	11 (14.9)	15 (16.0)	0.86
** Thick meconium-stained**	39 (23.2)	8 (10.8)	31 (33.0)	**<0.001**
**Vaginal delivery** (n, %)	81 (47.4)	36 (48.0)	45 (46.9)	0.88
** Total postpartum blood loss** (mL; median, IQR)	425 (300–712)	500 (300–700)	400 (300–750)	0.73
** Postpartum hemorrhage** (n, %)	54 (31.6)	24 (32.0)	30 (31.3)	0.91
**Intrapartum chorioamnionitis details**				
** Maximum maternal temperature** (°C; median, IQR)	38.0 (37.7–38.3)	38.0 (37.8–38.2)	38 (37.7–38.3)	0.82
** Intrapartum maternal leukocyte count** (×1000/μL; median, IQR)	17.7 (15.6–21.0)	17.3 (15.1–19.8)	18.4 (16.1–21.6)	**0.04**
** Maternal leukocytosis ≥ 15,000** (μL; n, %)	132 (81.5)	58 (79.5)	74 (83.1)	0.54
** Intrapartum maternal heart rate** (bpm; median, IQR)	93 (85–105)	90 (83–104)	95 (85–105)	0.45
** Maternal heart rate ≥ 100 bpm** (n, %)	49 (42.2)	18 (38.3)	31 (44.9)	0.48
** Maternal plasma CRP** (mg/L; median, IQR)	27 (14.7–53.2)	23 (12.5–47.5)	34 (15.5–62.5)	0.06
** Maternal lactate level** (mmol/L; median, IQR)	1.6 (0.9–2.5)	1.2 (0.8–2.1)	1.8 (0.9–2.8)	0.16
** Fetal tachycardia** (>160 bpm) (n, %)	106 (62)	46 (61.3)	60 (62.5)	0.87
** Antepartum maternal antibiotic administration** (n, %)	147 (86)	63 (84.0)	84 (87.5)	0.51
**Maternal postpartum outcomes**				
** Persistent postpartum maternal fever** (n, %)	18 (10.5)	4 (5.3)	14 (14.6)	0.05

IQR: interquartile range; BMI: body mass Index; PROM: premature rupture of membranes; GBS: Group B streptococcus; CRP: C-reactive protein.

**Table 2:** Factors independently associated with severe histological chorioamnionitis in the multivariable logistic regression model.

	Odds Ratio	95% Confidence Interval		*p* Value
**Maternal age** (y)	1.14	1.01	1.29	**0.024**
**Gestational age at delivery** (weeks)	1.86	0.99	3.49	0.051
**Duration of PROM**	1.00	0.99	1.00	0.12
**Maximum maternal temperature**	1.08	0.28	4.12	0.90
**Intrapartum maternal leukocyte count**	0.98	0.85	1.12	0.76
**Maternal plasma CRP**	1.00	0.99	1.02	0.28
**Fetal tachycardia (>160 bpm)**	1.92	0.53	6.98	0.32
**Labor analgesia**	0.80	0.20	3.20	0.75
**Amniotic fluid**				
**Clear**	-	-		
**Thin meconium-stained**	0.23	0.03	1.43	0.11
**Moderate meconium-stained**	1.21	0.25	5.80	0.81
**Thick meconium-stained**	5.88	1.12	30.86	**0.035**

**Table 3:** Factors independently associated with severe histological chorioamnionitis accompanied by NICU admission in the multivariable logistic regression model.

	Odds Ratio	95% Confidence Interval		*p* Value
**Maternal age** (y)	1.14	1.02	1.28	**0.015**
**Gestational age at delivery** (weeks)	1.14	0.67	1.93	0.61
**Duration of PROM**	1	0.99	1.00	0.26
**Intrapartum maternal leukocyte count**	1.05	0.93	1.18	0.40
**Fetal tachycardia (>160 bpm)**	3.10	1.02	10.59	**0.044**
**Amniotic fluid**				
**Clear**	-	-	-	-
**Thin meconium-stained**	0.80	0.13	3.62	0.78
**Moderate meconium-stained**	3.90	0.81	20.1	0.88
**Thick meconium-stained**	7.16	2.03	29.99	**0.0018**

## Data Availability

The data presented in this study are available on request from the corresponding author.

## Supplementary Materials

The following supporting information can be downloaded at https://www.mdpi.com/article/10.3390/medicina62050937/s1, Supplementary Table S1: Neonatal outcomes: overall and stratified based on the severe histological confirmation of chorioamnionitis. Supplementary Table S2: Maternal demographic and obstetrics characteristics of the study population, overall and stratified based on the concomitant severe histologic chorioamnionitis accompanied by adverse neonatal course (NICU admission).
