# Supplementary material for: Predictors of Severe Histological Chorioamnionitis and Associated Neonatal Outcomes in Term Intrapartum Clinical Chorioamnionitis: A Retrospective Cohort Study
Source: Medicina (Kaunas). 2026 May 11;62(5):937. doi: 10.3390/medicina62050937 (PMC13208483; doi:10.3390/medicina62050937)
Supplement: Supplementary file 1 [file medicina-62-00937-s001.zip › medicina-4259666-supplementary.pdf]

medicina

MDPI

Supplementary material

Supplementary Table S1. Neonatal outcomes: overall and stratified based on the severe histological confirmation of chorioamnionitis.

	**Total**<br>**N= 171**	**Without severe acute histological chorioamnionitis**<br>**N= 75**	**With severe acute histological chorioamnionitis**<br>**N= 96**	**p value**
**Neonatal birthweight** (median, IQR)	3350 (3125-3682)	3300 (3097-3605)	3370 (3132-3700)	0.50
**Neonatal arterial pH** (median, IQR)	7.25 (7.18-7.29)	7.24 (7.19-7.29)	7.25 (7.17-7.29)	0.88
**Neonatal arterial BE** (median, IQR)	-4.9 (-7.8 - -2.3)	-4.9 (-7.0 - -3.1)	-5.0 (-8.2 - -2.1)	0.92
**5' Apgar < 7** (n, %)	16 (9.5)	4 (5.4)	12 (12.8)	0.11
**Neonatal acidosis** (n, %)	5 (3.1)	1 (1.4)	4 (4.4)	0.38
**Signs and symptoms suspicious of early neonatal sepsis** (n, %)	87 (52.1)	36 (48.6)	51 (54.8)	0.43
**Neonatal leucocytes** (median, IQR)	21.33 (17.01-25.47)	21.09 (17.45-25.91)	21.33 (16.94-25.38)	0.72
**NICU admission** (n, %)	53 (31.2)	16 (21.3)	37 (38.9)	**0.01**
**Duration of NICU stay,** (days; median, IQR)	4 (2-7)	2 (1-4)	6 (2-8)	**0.008**
**Positive neonatal blood culture**	4 (4.5)	1 (1.1)	3 (3.4)	0.63
**Artificial ventilation – labor and delivery unit**	43 (25.3)	17 (22.7)	26 (27.4)	0.48
**Artificial ventilation - NICU**	24 (14.3)	7 (9.5)	17 (18.1)	0.11
**Neonatal acidosis (pediatric criteria)**	11 (6.5)	4 (5.3)	7 (7.4)	0.76
**Confirmed neonatal infection**	22 (14.7)	8 (12.5)	14 (16.3)	0.51
**Neonatal sepsis**	5 (2.9)	2 (2.7)	3 (3.2)	1.0
**RDS**	31 (18.2)	13 (17.3)	18 (18.9)	0.78
**Meconium aspiration**	18 (10.6)	5 (6.7)	13 (13.7)	0.14

**IVH/leukomalacia**	0 (0)	0 (0)	0 (0)	1
**Retinopathy of prematurity**	0 (0)	0 (0)	0 (0)	1
**Necrotizing enterocolitis**	0 (0)	0 (0)	0 (0)	1
**Neonatal antibiotic therapy**	36 (21.2)	11 (14.7)	25 (26.3)	0.06
**Neonatal death**	0 (0)	0 (0)	0 (0)	1

BE: base excess; CRP: C-reactive protein; IQR: interquartile range; NICU: neonatal intensive care unit; RDS: respiratory distress syndrome; UA: umbilical artery; UV: umbilical vein, IVH: intraventricular haemorrhage.

Supplementary Table S2. Maternal demographic and obstetrics characteristics of the study population, overall and stratified based on the concomitant severe histologic chorioamnionitis accompanied by adverse neonatal course (NICU admission).

	**Total**<br>**N= 171**	**Women without combination of NICU admission and acute severe histological chorioamnionitis**<br>**N= 134**	**Women with severe histologic chorioamnionitis accompanied by adverse neonatal course (NICU admission).**<br>**N= 37**	**P value**
**Maternal demographic and obstetrics characteristics**				
**Maternal age** (y; median, IQR)	31 (29-35)	31 (28-35)	33 (29.5-39)	**0.013**
**Pre-pregnancy BMI** (kg/m^2; median, IQR)	22 (20-25)	22 (20-25)	22 (20-25.2)	0.98
**Nulliparity** (n, %)	150 (87.7)	119 (88.8)	31 (83.8)	0.40
**Infertility treatments** (n, %)	14 (8.2)	10 (7.5)	4 (10.8)	0.51
**Gestational age at delivery** (weeks; median, IQR)	40.3 (39.6-41.1)	40.2 (39.2-41.1)	40.6 (39.9-41.3)	0.12
**Recto-vaginal GBS colonization** (n, %)	30 (17.8)	27 (20.3)	3 (8.3)	0.096
**Preexisting diabetes mellitus** (n, %)	1 (0.6)	0 (0)	1 (2.7)	0.22

**Gestational diabetes mellitus** (n, %)	22 (12.9)	18 (13.4)	4 (10.8)	0.79
**Labor characteristics**				
**Induction of labor** (n, %)	84 (49.1)	70 (52.2)	14 (37.8)	0.12
**Labor analgesia** (n, %)	127 (74.7)	102 (76.1)	25 (69.4)	0.41
**Duration of labor analgesia** (min; median, IQR)	480 (240-632)	480 (340-618)	508 (349-669)	0.62
**PROM** (n, %)	113 (67.7)	85 (64.9)	28 (77.8)	0.14
**Duration of PROM** (min; median, IQR)	593 (353-900)	586 (322-840)	733 (389-1140)	0.27
**PROM ≥24 hours** (n, %)	10 (9.1)	8 (9.5)	2 (7.7)	1.0
**Duration of labor, I stage** (min; median, IQR)	330 (180-480)	330 (185-476)	360 (155-510)	0.90
**Duration of labor, II stage** (min; median, IQR)	85 (45-126)	90 (45-120)	75 (46-133)	0.90
**Total duration of labor** (min; median, IQR)	410 (265-575)	404 (272-570)	473 (225-655)	0.78
**Amniotic fluid** (n, %)				**0.01**
**Clear**	88 (55.4)	77 (57.9)	11 (31.4)	**0.003**
**Thin meconium-stained**	15 (8.9)	13 (9.8)	2 (5.7)	0.41
**Moderate meconium-stained**	26 (15.5)	18 (13.5)	8 (22.9)	0.22
**Thick meconium-stained**	39 (23.2)	25 (18.8)	14 (40.0)	**0.014**
**Intrapartum chorioamnionitis details**				
**Maximum maternal temperature** (°C; median, IQR)	38.0 (37.7-38.3)	38 (37.7-38.2)	38.0 (37.4-38.4)	0.70
**Intrapartum maternal leukocyte count** (x 1000/µL; median, IQR)	17.3 (15.1-19.8)	17.5 (15.5-20.9)	18.4 (16.2-21.2)	0.44
**Maternal leukocytosis ≥ 15,000** (µL; n, %)	132 (81.5)	105 (81.4)	27 (81.8)	0.96
**Intrapartum maternal heart rate** (bpm; median, IQR)	93 (85-105)	93 (83-105)	93 (88-104)	0.47

**Maternal heart rate ≥100 bpm** (n, %)	49 (42.2)	38 (42.2)	11 (42.3)	0.99
**Maternal plasma CRP** (mg/L; median, IQR)	27 (14.7-53.2)	25.0 (14.0-50.5)	40.0 (18.5-58.0)	0.13
**Maternal lactate level** (mmol/L; median, IQR)	1.6 (0.9-2.5)	1.6 (0.9-2.8)	1.6 (0.1-1.9)	0.31
**Fetal tachycardia (>160 bpm)** (n, %)	106 (62)	82 (61.2)	24 (64.9)	0.68
**Antepartum maternal antibiotic administration** (n, %)	147 (86)	113 (84.3)	34 (91.9)	0.24
IQR: interquartile range; BMI: body mass Index; PROM: premature rupture of membranes; GBS: Group B Streptococcus; CRP: C-reactive protein; NICU: neonatal intensive care unit.				
